# cGAS‒STING signaling and function in metabolism and kidney diseases

**DOI:** 10.1093/jmcb/mjab066

**Published:** 2021-10-19

**Authors:** Juli Bai, Feng Liu

**Affiliations:** 1 Department of Pharmacology, University of Texas Health at San Antonio, San Antonio, TX 78229, USA; 2 National Clinical Research Center for Metabolic Diseases, Metabolic Syndrome Research Center, Key Laboratory of Diabetes Immunology, Ministry of Education, and Department of Metabolism and Endocrinology, The Second Xiangya Hospital of Central South University, Changsha 410011, China

**Keywords:** cGAS, STING, obesity, NAFLD, NASH, AKI, CDK

## Abstract

The cyclic GMP‒AMP synthase (cGAS)‒stimulator of interferon genes (STING) signaling pathway senses the presence of cytosolic DNA and, in turn, triggers downstream signaling to induce the expression of inflammatory and type I interferon genes in immune cells. Whereas the innate immune function of the cGAS‒STING pathway is well studied over the past years, emerging evidence suggests that this signaling pathway may have additional functions beyond innate immune surveillance. Consistent with this notion, dysregulation of the cGAS‒STING signaling pathway in adipocytes, hepatocytes, and renal proximal tubule epithelial cells are associated with metabolic dysfunction, impaired energy homeostasis, and kidney diseases. In this review, we summarize current understanding of the cGAS‒STING pathway in several metabolic diseases such as obesity, insulin resistance, alcoholic and nonalcoholic fatty liver diseases, as well as acute kidney injury and chronic kidney disease. We also review the interaction between the cGAS‒STING pathway and lipid metabolism. Lastly, we discuss potential mechanisms by which cGAS‒STING signaling regulates metabolism and point toward future avenues of research targeting the cGAS‒STING pathway as possible means to treat common metabolic disorders.

## Introduction

The cyclic GMP‒AMP (cGAMP) synthase (cGAS) is a cytosolic DNA sensor that plays a vital role in innate immune defense against pathogen infection or damage-induced host DNA aberrantly localized in the cytosol (Hopfner and Hornung, 2020). Activation of cGAS increases cellular levels of 2ʹ3ʹ-cGAMP, which triggers stimulator of interferon genes (STING)-dependent inflammatory and type I interferon (IFN) gene production. The cGAS‒STING signaling pathway has received considerable attention over the past years due to its pivotal roles not only in innate immune surveillance but also in many other previously unrecognized biological functions, e.g. regulating nonalcoholic or alcoholic liver disease (Bai et al., 2017; Luo et al., 2018; Yu et al., 2019), fat tissue thermogenesis (Bai et al., 2020), kidney diseases (Allison, 2019; Chung et al., 2019; Maekawa et al., 2019), and neurodegeneration (Gamdzyk et al., 2020; Jauhari et al., 2020; Paul et al., 2021).

Human protein Atlas analysis reveals that cGAS is not only highly expressed in most of the immune-related tissues/organs, such as the spleen, lymph node, tonsil, and thymus but is also found in many nonimmune-related tissues such as the brain, lung, pancreas, kidney, urinary bladder, prostate, and, to a less extent, the liver, adipose tissue, and soft tissue (https://www.proteinatlas.org/ENSG00000164430-CGAS/tissue). We and others also detected cGAS expression in several metabolism-related tissues in mice including the epidydimal adipose tissue, inguinal adipose tissue, brown adipose tissue, liver, heart, and kidney (Bai et al., 2017, 2020; Cao et al., 2018; Luo et al., 2018; Maekawa et al., 2019; Xiong et al., 2019; Hu et al., 2020). Unlike cGAS, the expression of STING is more tissue selective and high levels of STING protein were detected in immune-related tissues such as the spleen, lymph node, tonsil, and, to a less extent, bone marrows (https://www.proteinatlas.org/ENSG00000184584-TMEM173/tissue). High STING protein expression was also detected in a number of other nonimmune tissues such as the lung, rectum, pancreas, testis, prostate, urinary bladder, heart muscle, and adipose tissue, but little STING expression was detected in the liver, smooth muscle, or skeletal muscle. These results are consistent with the finding that human or mouse hepatocytes do not express STING (Thomsen et al., 2016; Lei et al., 2018; Yu et al., 2019), though some other studies show that low levels of functional STING are present in hepatocytes (Iracheta-Vellve et al., 2016; Qiao et al., 2018). While further investigation is needed to clarify this discrepancy, the finding that cGAS and STING are expressed in metabolism-related cells suggests a potential role of the cGAS‒STING signaling pathway in regulating metabolism. However, whereas the role of the cGAS‒STING pathway in innate immunity is well studied and extensively reviewed, much less is known about its function in other cellular events that are not directly related to innate immune surveillance. In the current review, we discuss recent development on the functions and regulation of the cGAS‒STING signaling pathway, focusing mainly on its roles in metabolism, energy homeostasis, and kidney function.

## Signaling and regulation of cGAS in innate immunity

**Figure 1:**
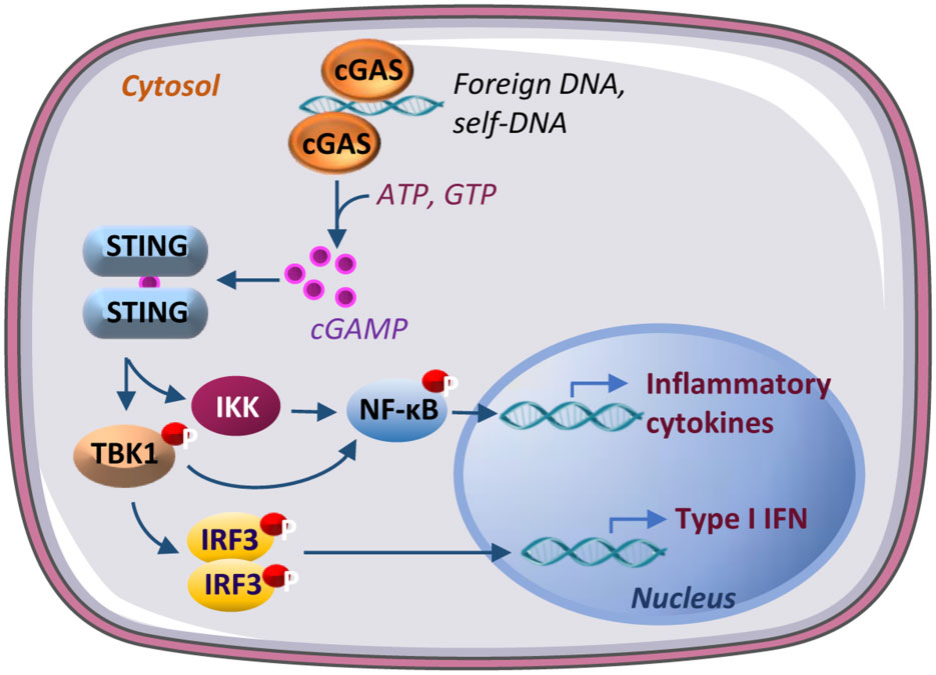
The cGAS‒STING pathway in inflammatory and IFN responses. cGAS is activated upon detecting foreign DNA or self-DNA aberrantly localized in the cytosol. Activated cGAS uses ATP and GTP as substrates to synthesize the second messenger cGAMP, which binds to and activates STING. STING recruits TBK1, which in turn autophosphorylates itself and IRF3, leading to the production of type I IFN. Activated STING also activates IKK, which promotes NF-κB nuclear translocation-induced gene expression of inflammatory cytokines.

The innate immune system provides immediate and rapid defenses against pathogens and is the dominant host defense system in most organisms. An important strategy by which the innate immune system recognizes pathogens is to detect pathogen-derived DNA in the cytosol, which is mediated by the cytosolic DNA sensor cGAS (Li et al., 2013; Sun et al., 2013; Wu et al., 2013). The binding of double-stranded DNA (dsDNA) activates cGAS, resulting in the synthesis of the second messenger cGAMP. cGAMP interacts with STING and promotes STING translocation from the endoplasmic reticulum (ER) to the ER–Golgi intermediate compartment and the Golgi, which activates the TANK binding kinase 1 (TBK1)‒interferon regulatory factor 3 (IRF3)‒type I IFN signaling pathway (Hopfner and Hornung, 2020). The binding of cGAMP to STING also activates IκB kinase (IKK), which increases the phosphorylation and activation of nuclear factor-κB (NF-κB) and consequently the production of many inflammatory cytokines (Shu and Wang, 2014). In addition to pathogen-derived dsDNA, cGAS is also activated by self-DNA, like mitochondrial DNA (mtDNA) and nuclear DNA that are aberrantly localized in the cytosol or in the micronuclei under pathophysiological conditions, including mitochondrial stress, genomic instability, and/or DNA damage, leading to sterile inflammation associated with various diseases such as autoimmune diseases, cancers, and metabolic dysfunctions (Li and Chen, 2018; Bai and Liu, 2019; Figure 1). Thus, the cGAS‒STING pathway may act as a double-edged sword that, on the one hand, offers the first line of defense against infection, but on the other hand, causes detrimental consequences to the system, such as cancer and metabolic dysfunction, when inappropriately activated.

The activity and function of cGAS are spatially and temporally regulated in cells and compartmentalization has been recognized as a major mechanism that prevents cGAS from activation by self-DNA such as mtDNA and nuclear DNA, which are sequestered from the cytosol by mitochondrial membranes or nuclear envelope (Gekara and Jiang, 2019). However, while cGAS was initially identified as a cytosolically localized DNA sensor (Gao et al., 2013; Sun et al., 2013), subsequent studies show that this enzyme is also localized in the nucleus in a number of cells (Orzalli et al., 2015; Lahaye et al., 2018; Liu et al., 2018; Zhong et al., 2020; Sun et al., 2021). Some reports show that endogenous cGAS is predominantly localized in the nucleus where it is tethered tightly with chromatin (Jiang et al., 2019; Volkman et al., 2019). Cryo-electron microscopy studies indicate that cGAS binds to the acidic patch of the heterodimer histone H2A‒H2B in nucleosomes, which greatly blocks cGAS dimerization and thus prevents the enzyme from activation by nuclear DNA (Zierhut et al., 2019; Boyer et al., 2020; Kujirai et al., 2020; Michalski et al., 2020; Pathare et al., 2020; Wang et al., 2020a; Zhao et al., 2020; Bai and Liu, 2021). In addition to the cytosol and nucleus, cGAS has also been found to reside on the plasma membrane in human and mouse macrophages, which has been suggested as a mechanism that prevents cGAS from activation by self-DNA in the cytosol under conditions of genotoxic stress (Barnett et al., 2019). Taken together, these findings suggest that subcellular localization plays a critical role in regulating cGAS activity and function. In addition to cellular compartmentalization, cGAS activity and function are also regulated by various posttranslational modification mechanisms including phosphorylation (Seo et al., 2015; Song et al., 2020; Zhong et al., 2020;  Li et al., 2021), acetylation (Dai et al., 2019; Song et al., 2020), ubiquitination (Wang et al., 2017), glutamylation (Xia et al., 2016), and SUMOylation (Hu et al., 2016). Excellent reviews have been published recently with more details (Wu and Li, 2020; Yu and Liu, 2021).

## cGAS‒STING signaling in metabolism

### Activation of the cGAS‒STING signaling pathway promotes insulin resistance in adipose tissue

**Figure 2:**
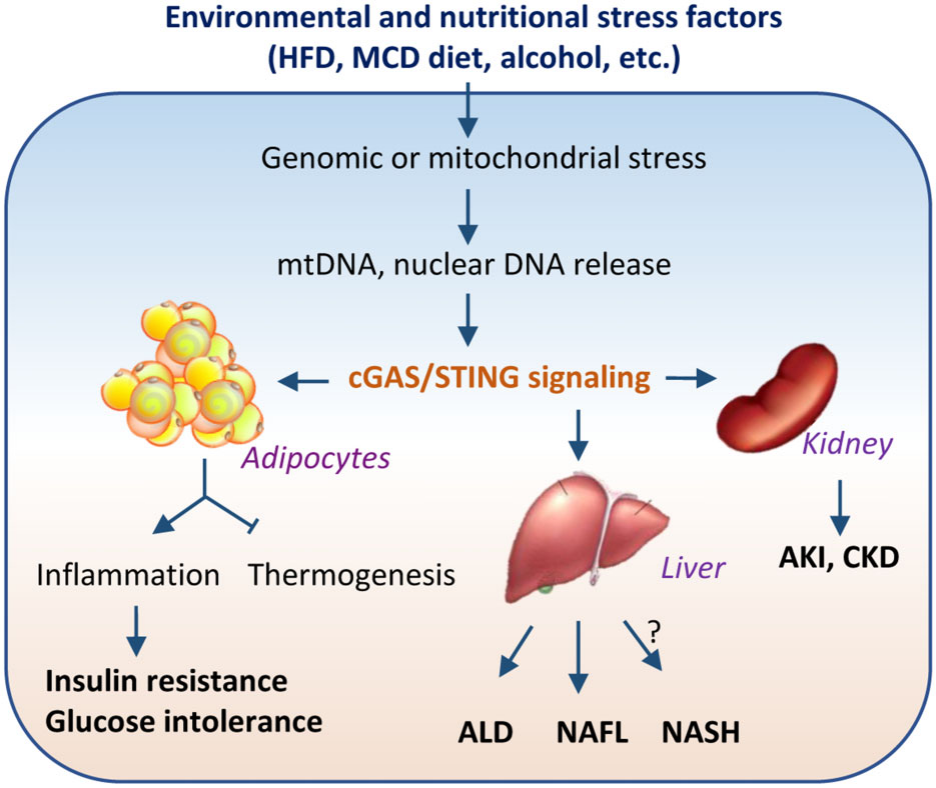
The cGAS‒STING pathway in metabolic and kidney diseases. HFD, MCD diet, or alcohol induces the activation of the cGAS‒STING pathway in metabolic tissues such as adipose tissue and the liver. In adipose tissue, activated cGAS‒STING pathway promotes inflammation and suppresses thermogenesis, resulting in obesity and insulin resistance in mice. In the liver, cGAS‒STING activation is associated with the development of ALD, NAFL, and NASH. In the kidney, activated cGAS‒STING pathway contributes to both AKI and CKD.

The immune system is tightly linked to metabolic regulation in all animals, and proper redistribution of energy is crucial during immune challenges (Odegaard and Chawla, 2015; Alwarawrah et al., 2018). In addition to a central role in innate immune defense against pathogen infection, recent studies also suggest a critical role of the cGAS‒STING pathway in metabolism. Obesity triggers a state of chronic, low-grade inflammation in metabolism-related tissues such as the liver, muscle, and fat, leading to insulin resistance and metabolic diseases, e.g. type 2 diabetes (Wu and Ballantyne, 2020). While numerous studies strongly suggest that sterile inflammation plays a major role in obesity-induced insulin resistance and various metabolic diseases, the precise underlying mechanisms remain uncertain. Evidence accumulated over the past years reveals that activation of the cGAS‒STING pathway by self-DNA may contribute to obesity-induced sterile inflammation and associated metabolic dysfunctions (Figure 2; Bai and Liu, 2019). Under high-fat diet (HFD) feeding condition, mouse adipose tissue displayed increased cytosolic mtDNA levels and activation of the cGAS‒STING signaling pathway in both adipocytes and adipose tissue stromal vascular fractions including macrophages, which correlated with increased inflammation (Bai et al., 2017). Increasing mitochondrial stress by adipocyte-specific knockout of the mitochondrial chaperone protein disulfide-bond A oxidoreductase-like protein (DsbA-L) activated the cGAS‒STING pathway and promoted inflammatory responses and type I IFN gene expression (Bai et al., 2017). Conversely, reducing mtDNA release by adipocyte-specific overexpression of DsbA-L alleviated HFD-induced activation of the cGAS‒STING pathway and protected mice against HFD-induced inflammation and insulin resistance (Bai et al., 2017). Consistent with these findings, STING deficiency partially prevented HFD-induced adipose tissue inflammation, insulin resistance, and glucose intolerance (Mao et al., 2017). In addition, activation of the STING downstream signaling molecule IRF3, whose expression is highly induced in adipocytes of obese mice and humans, led to insulin resistance in adipocytes (Kumari et al., 2016). On the other hand, ablation of IRF3 in mice reduced HFD-induced macrophage infiltration into fat pads, inflammatory gene expression, and insulin resistance (Kumari et al., 2016). Furthermore, activation of virus-induced type I interferon (IFNα/β) receptor (IFNAR) signaling in adipocytes promoted inflammatory potential of adipocytes and potentially exacerbated metabolic disorders in mice (Chan et al., 2020). Taken together, these results suggest that abnormal activation of cGAS‒STING‒IFN signaling in adipose tissue contributes to obesity-induced insulin resistance and metabolic dysfunction. However, the role of the STING downstream signaling component TBK1 in metabolism in adipose tissue is less straightforward. The expression and activity of TBK1 are induced in adipose tissues during diet-induced obesity (Chiang et al., 2009; Reilly et al., 2013; Zhao et al., 2018), suggesting a potential involvement of this kinase in obesity-induced metabolic dysfunction. Surprisingly, adipose TBK1 deficiency exaggerates inflammation and insulin resistance, demonstrating a beneficial role of adipose TBK1 in metabolism (Zhao et al., 2018). Further investigation shows that TBK1 is able to inhibit NF-κB activity via phosphorylation and degradation of NF-κB interacting kinase, and thus its deficiency leads to increased NF-κB activation and inflammation (Zhao et al., 2018). Given that TBK1 is not only activated by cGAS signaling but also by many other cellular factors such as lipopolysaccharide, cytokines (e.g. TNFα), and nutrition status (Zhao and Saltiel, 2020), its contribution to cGAS‒STING signaling-induced metabolic alteration in adipose tissue remains to be further elucidated.

### Activation of cGAS‒STING signaling in adipose tissue promotes obesity by suppressing thermogenesis

In addition to regulating insulin sensitivity and action, activation of the cGAS‒STING pathway has also been implicated in regulating energy homeostasis (Bai et al., 2020). Obesity is caused by excessive energy intake and/or inefficient energy expenditure, partially due to impaired thermogenic function of brown or beige fat, which normally dissipates energy as heat through nonshivering thermogenesis by the high density of mitochondria. Fat-specific DsbA-L knockout mice displayed higher mitochondrial stress and enhanced activation of the cGAS‒STING pathway in adipose tissue, concurrently with decreased thermogenic gene expression in response to cold exposure, reduced energy expenditure, and increased body weight (Bai et al., 2020). Conversely, inactivation of STING or reduction of mitochondrial stress by fat-specific overexpression of DsbA-L protected mice against HFD-induced obesity. Suppressing cGAS or STING expression by shRNA or inhibiting the activity of the STING downstream kinase TBK1/IKKε greatly increased protein kinase A (PKA) phosphorylation and uncoupling protein 1 gene expression in DsbA-L-deficient adipocytes, demonstrating a cell-autonomous effect of the cGAS‒STING signaling pathway on thermogenic gene expression (Bai et al., 2020). Treating primary adipocytes with 2ʹ3ʹ-cGAMP, which activates STING downstream signaling, increased the phosphodiesterase PDE3B/PDE4 activity and reduced intracellular cAMP levels. On the other hand, knockout of STING significantly increased basal and the β3-adrenoceptor agonist CL316243-induced cAMP levels in primary adipocytes (Bai et al., 2020). These results demonstrate that, by inhibiting cAMP‒PKA signaling and thermogenic gene expression, mitochondrial stress-activated cGAS‒STING pathway functions as a sentinel signal that suppresses thermogenesis in adipose tissue (Figure 2). Consistent with a negative role of adipose cGAS‒STING signaling in regulating thermogenesis, adipose-specific knockout of TBK1 attenuates HFD-induced obesity by increasing energy expenditure, which has been attributed to increased AMPK activity that enhances catabolism (Zhao et al., 2018). Treating obese mice with amlexanox, a TBK1 and IKKε inhibitor, elevates energy expenditure through increasing thermogenesis (Reilly et al., 2013). The TBK1 downstream target IRF3 has been shown to be a strong repressor of thermogenic gene expression and oxygen consumption in adipocytes (Yan et al., 2021), and ablation of IRF3 in mice promoted beige fat development and increased energy expenditure (Kumari et al., 2016). In addition, ectopic activation of type I IFN signaling in brown adipocytes caused profound mitochondrial dysfunction and reduced thermogenic capacity (Kissig et al., 2017). These findings reveal that adipose cGAS‒STING‒IFN signaling negatively regulates thermogenesis and energy expenditure. Nevertheless, further studies using adipose tissue-specific cGAS and STING knockout mice would be necessary to verify the roles of adipose cGAS and STING in the regulation of energy homeostasis.

### The role of cGAS‒STING signaling in the pathogenesis of nonalcoholic and alcoholic fatty liver diseases

Nonalcoholic fatty liver (NAFL), which is characterized by accumulation of excess fat in the liver of people who consume little or no alcohol, contributes to the development of nonalcoholic steatohepatitis (NASH) and potentially cirrhosis and hepatocellular carcinoma (Vos and van de Sluis, 2021). Increased expression of STING is detected in the liver of NAFL (Luo et al., 2018) and NASH (Wang et al., 2020b) human patients compared with the respective control, especially in the liver portal tract of NASH patients with fibrosis compared to that of healthy controls. Elevated expression levels of cGAS‒STING signaling components such as cGAS, STING, and IRF3 are also found in the liver of HFD-fed (Qiao et al., 2018) or NASH mouse models (Luo et al., 2018; Xiong et al., 2019), suggesting that activation of the cGAS‒STING pathway contributes to diet-induced NAFL or NASH. HFD- or methionine- and choline-deficient (MCD) diet-induced steatosis, fibrosis, and inflammation are attenuated in STING-deficient mice (Luo et al., 2018; Yu et al., 2019). Knocking down either STING or IRF3 also significantly reduced free fatty acid-induced hepatic inflammation and apoptosis in human hepatic LO-2 cells, as evidenced by the downregulation of the NF-κB signaling pathway, reduced expression of inflammatory cytokines, and decreased apoptotic signaling (Qiao et al., 2018). STING expression is mainly found in macrophages, including monocyte-derived macrophages (CCR2^+^, S100A9^+^), Kupffer cells (CD68^+^), and CD163^+^ macrophages (Wang et al., 2020b). Transplantation of bone marrow cells from STING knockout mice to irradiated wild-type mice decreased HFD-induced hepatic steatosis compared with that in HFD-fed mice transplanted with bone marrow cells from wild-type mice. On the other hand, transplantation of bone marrow cells from wild-type mice to STING knockout mice restored HFD-induced severity of steatosis and inflammation, suggesting a major role of the macrophage STING in diet-induced metabolic dysfunction in the liver (Luo et al., 2018). Nevertheless, the tissue-specific roles of cGAS and other cGAS‒STING signaling components in NAFL and NASH remain to be determined.

In addition to playing a role in nonalcoholic fatty liver disease (NAFLD) development, activation of the cGAS‒STING signaling pathway has also been shown to contribute to liver injury in alcohol-related liver disease (ALD) (Petrasek et al., 2013; Luther et al., 2020). The expression of the cGAS‒STING pathway components such as cGAS, STING, TBK1, and IRF3 positively correlated with ALD disease severity in humans and alcohol-fed mice. In addition, knockout of cGAS and IRF3 protected mice against ALD (Luther et al., 2020). The effect of cGAS‒STING signaling on ALD is facilitated by connexin 32 (Cx32), the predominant hepatic gap junction that promotes intercellular transfer of 2ʹ3ʹ-cGAMP from injured to bystander cells (Patel et al., 2009; Ablasser et al., 2013). In agreement with this finding, knockout of Cx32 in mice alleviated alcohol-induced activation of the cGAS‒STING signaling pathway, inflammation, and hepatocyte injury (Luther et al., 2020). These findings demonstrate a key role of the cGAS‒STING pathway in ALD pathogenesis, highlighting an attractive therapeutic target for combating ALD.

### cGAS‒STING signaling and lipid metabolism

Dysregulation in cholesterol and fatty acid synthesis and/or their cellular uptake leads to various metabolic disorders such as NAFLD (Malhotra et al., 2020). The synthesis of fatty acids and cholesterol is regulated by several basic helix‒loop‒helix leucine zipper transcription factors such as sterol regulatory element-binding protein 1 (SREBP1) and SREBP2, respectively (Madison, 2016; Shimano and Sato, 2017; Luo et al., 2020). SREBPs are synthesized as precursors in the ER and processed to mature nuclear forms in Golgi apparatus before they become the functional transcription factors (Brown and Goldstein, 1997). The translocation of SREBPs from the ER to the Golgi depends on their interaction with SREBP cleavage-activating protein (SCAP), an ER sterol-sensing protein that chaperones SREBPs to the Golgi through binding to COPII-coated vesicles (Luo et al., 2020).

**Figure 3:**
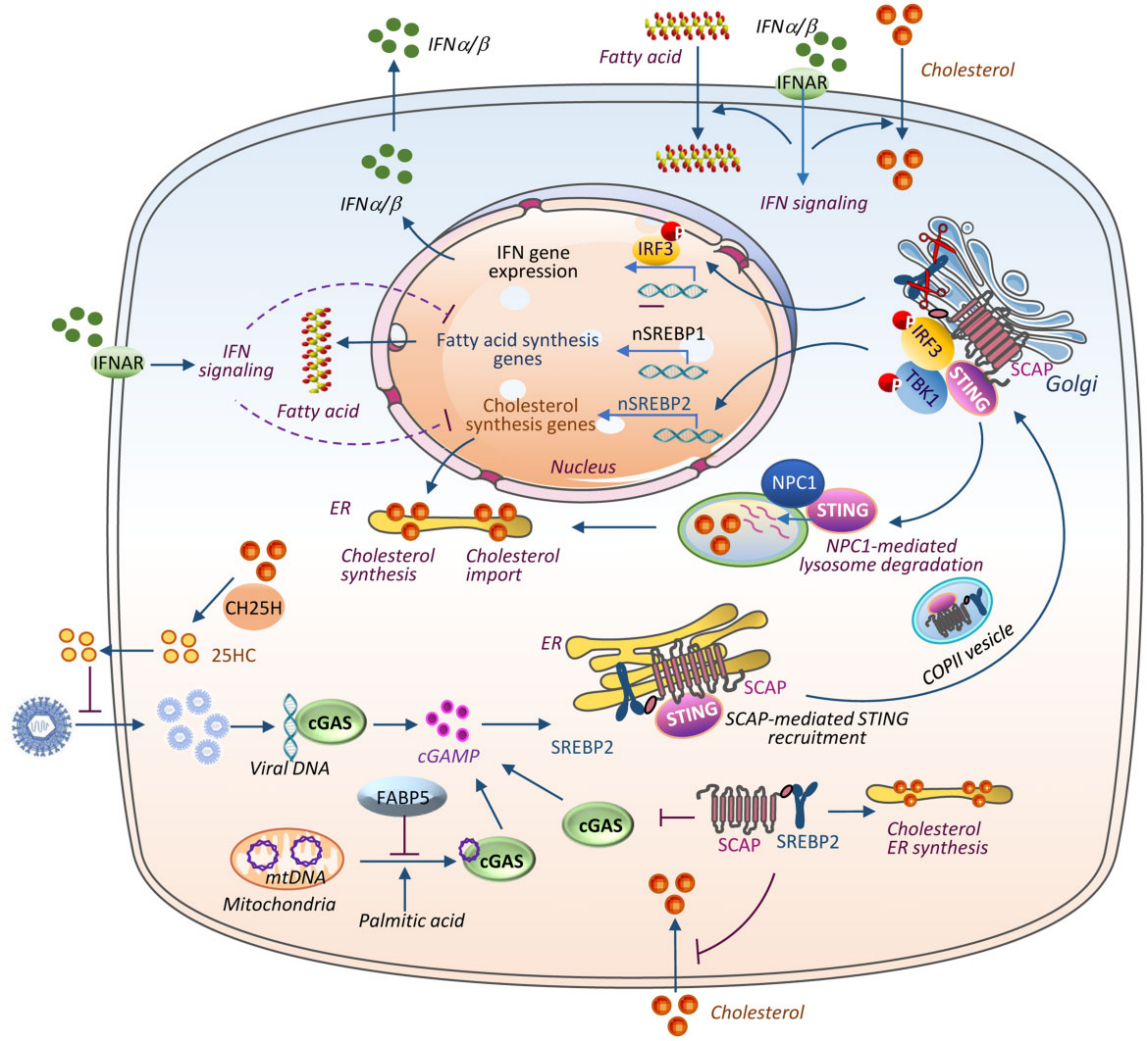
cGAS‒STING signaling and lipid metabolism. IFNα/β‒IFNAR signaling shifts the balance of fatty acid/sterol homeostasis by suppressing the synthesis and increasing the import from microenvironment. Genetically enforcing this metabolic shift by knocking out SREBP2 or SCAP, but not SREBP1, primes cGAS‒STING-induced type I IFN response and antiviral activity. Controversially, SCAP-mediated SREBP2 recruitment in the ER and trafficking to the Golgi promote STING-mediated IRF3 activation and antiviral response. The lysosomal membrane protein NPC1 recruits STING to the lysosome for degradation. NPC1 knockout primes and boosts STING signaling by two means: first, it blocks cholesterol export from the lysosome to the ER, which induces physical tethering of STING to SCAP‒SREBP2 for trafficking from the ER to the Golgi and consequent activation; second, it induces STING accumulation by lysosome-mediated degradation dysfunction. CH25H oxidizes cholesterol to produce 25HC, which serves as IFN-independent antiviral factor by broadly inhibiting viral entry. FABP5 knockout or palmitic acid treatment promotes mtDNA release into the cytosol, where it activates the DNA sensor cGAS-dependent downstream signaling. nSREBP, nuclear SREBP.

Accumulated evidence reveals that lipid metabolism and type I IFN response are coregulated, which enables innate immune cells to coordinate with metabolic changes required for antiviral responses (York et al., 2015; Chen et al., 2016; Chu et al., 2021). In support of this view, infecting bone marrow-derived macrophages (BMDMs) with a dsDNA virus, the murine gammaherpesvirus-68, or treating these cells with IFNβ or the toll-like receptor 3 ligand poly I:C, which greatly enhances IFN-stimulated gene signature, significantly suppressed the synthesis of cholesterol as well as saturated and unsaturated long-chain fatty acids while increasing the import of fatty acids and cholesterol through an IFNAR signaling-dependent mechanism (York et al., 2015). These findings demonstrate that activation of innate immune response disturbs lipid metabolism balance (York et al., 2015). Interestingly, numerous studies suggest that the regulation of the innate immune pathway and the pathways controlling cholesterol synthesis is reciprocal (Kwak et al., 2000; Weitz-Schmidt et al., 2001; Castrillo et al., 2003; Blanc et al., 2013). Consistent with this notion, deleting or silencing SCAP or SREBP2, which shifts the balance from cholesterol synthesis to cholesterol import, induced cGAS‒STING-mediated type I IFN response and enhanced resistance to viral infection in BMDMs and mouse embryonic fibroblasts (York et al., 2015). In contrast, cholesterol supplementation reduced the expression of IFN-induced genes and abrogated the antiviral phenotype in SREBP2- or SCAP-deficient cells (York et al., 2015). However, several studies reported that SREBP2 trafficking by SCAP primes STING cellular translocation to activate its downstream signaling (Chen et al., 2016; Chu et al., 2021). Chen et al. (2016) found that SCAP interacts directly with STING and serves as a scaffold adaptor to recruit IRF3 to the perinuclear/Golgi foci (also called perinuclear microsome), thus activating the antiviral IFN signaling (Figure 3). Silencing SCAP impairs STING-dependent IRF3-responsive gene expression and renders mice more susceptible to HSV-1 infection (Chen et al., 2016). Very recently, Chu et al. (2021) identified a lysosomal membrane protein Niemann‒Pick type C1 (NPC1) as a cofactor in the trafficking of STING to the lysosome for degradation in both human and mouse cells. NPC1 knockout primes and boosts STING signaling by two means: blocking cholesterol export from the lysosome to the ER, which induces physical tethering of STING to SCAP‒SREBP2 for trafficking from the ER to the Golgi and consequent activation, and inducing STING accumulation by lysosome-mediated degradation dysfunction (Chu et al., 2021; Figure 3). Activation of type I IFN signaling induces the expression of cholesterol-25-hydroxylase (CH25H), an enzyme that catalyzes oxidation of cholesterol to produce 25-hydroxycholesterol (25HC), which serves as IFN-independent antiviral factor that broadly inhibits viral entry (Liu et al., 2013; Figure 3). Taken together, these studies highlight a functional link between innate immunity and cholesterol metabolism.

In addition to cholesterol, perturbance of fatty acid metabolism by inhibiting fatty acid binding protein 5 (FABP5), a lipid chaperone that facilitates fatty acid uptake and intracellular lipid trafficking, also leads to the activation of cGAS‒STING-mediated type I IFN signaling (Field et al., 2020). Genetic or pharmacologic inhibition of FABP5 reduced the expression of genes involved in elongation and desaturation of fatty acids, decreased levels of cardiolipin species, and impaired mitochondrial function, leading to mtDNA release-induced activation of the cGAS‒STING innate immune pathway in regulatory T cells (Field et al., 2020). The involvement of fatty acid in the regulation of the cGAS‒STING pathway has also been reported by Yuan et al. (2017), who found that palmitic acid treatment-induced mtDNA release and activation of cGAS‒STING signaling, suggesting an enhanced antiviral capability by fatty acid supplementation (Figure 3). These findings suggest a tight association between the cGAS‒STING‒IFN response and fatty acid metabolism. Further studies will be needed to determine whether the reciprocally regulatory relationship between cGAS‒STING signaling and the lipid/sterol synthesis pathway holds in metabolic tissues such as the liver, a major site for fatty acid and cholesterol synthesis and metabolism, and whether the innate immune pathway functions as an important regulator in lipid homeostasis in these tissues.

## The role of the cGAS‒STING pathway in the pathogenesis of kidney diseases

Kidney, a mitochondria-enriched and metabolism-active organ, is the second highest consumer of molecular oxygen in the body at rest (Pagliarini et al., 2008). Under renal pathophysiological conditions, increased oxygen consumption due to oxidative stress and decreased electrolyte transport efficiency cause renal hypoxia, which leads to mitochondrial stress and aberrant tubular inflammation (Hansell et al., 2013; Franzen et al., 2016). However, while impaired mitochondrial function and tubular inflammation are well known to contribute to the pathogenesis of acute kidney injury (AKI) and consequent chronic kidney disease (CKD) (Forbes and Thorburn, 2018), the precise molecular mechanisms linking mitochondrial damage to AKI and CDK remain elusive.

Maekawa et al. (2019) used a genetically engineered animal model of cisplatin-induced AKI and cultured tubular cells to investigate the mechanism underlying mitochondrial damage-induced AKI. They found that administrating cisplatin, a platinum-containing agent that causes nephrotoxicity in humans (Arany and Safirstein, 2003), activated the cGAS‒STING pathway in mice as well as in immortalized HK-2 human proximal tubular cells and primary renal proximal tubule epithelial cells (RPTECs) via a mitochondrial injury-dependent mechanism. Upregulation of the cGAS‒STING pathway was also detected in the kidney specimens of AKI patients compared to non-AKI human subjects, revealing a human relevance of this signaling pathway to AKI. The cisplatin-induced tubular inflammation and AKI phenotypes were ameliorated in STING knockout mice and in cGAS- or STING-suppressed HK-2 cells and RPTECs (Maekawa et al., 2019). Consistent with these findings, activation of the cGAS‒STING pathway by mtDNA that is aberrantly localized in the cytosol induced an immune response that triggered fibrotic changes in TECs and in tubule-specific *Tfam*-knockout mice (Chung et al., 2019). In contrast, suppressing cGAS and/or STING expression reduced the expression of inflammatory genes in cultured cells and alleviated inflammation, TEC apoptosis, and fibrosis in tubule-specific *Tfam*-knockout mice. Genetic and pharmacologic inhibition of STING also attenuated renal inflammation, tubular injury, renal fibrosis, as well as mitochondrial dysfunction in mice with folic acid-induced kidney injury or cisplatin-induced AKI (Chung et al., 2019; Gong et al., 2021). In addition, overexpression of DsbA-L, a mitochondrial localized chaperon protein that is known to protect mitochondrial stress-induced mtDNA release and activation of the cGAS‒STING pathway in adipose tissue (Bai et al., 2017, 2020), ameliorates high glucose-induced tubular damage and prevents ectopic fat deposition and lipid-related kidney damage in diabetic nephropathy (Chen et al., 2019; Yang et al., 2019). However, proximal tubule-specific knockout of DsbA-L has also been shown to attenuate unilateral ureteral obstruction-induced tubulointerstitial fibrosis, renal cell apoptosis, and inflammation, which is mediated via suppression of the Hsp90/p53 and Smad3/CTGF axes (Li et al., 2020). Further studies will be needed to determine whether cGAS‒STING signaling is activated in proximal tubules of DsbA-L knockout mice.

In addition to AKI, activation of the cGAS‒STING pathway is also implicated in the development of CKD. CKD-induced oxidative stress has been found to prime the cGAS‒STING pathway to trigger type I IFN response in vascular smooth muscle cells (VSMCs), which are shown to be both type I IFN-responsive and type I IFN-productive cells (Bi et al., 2021). VSMCs could sense oxidative stress-induced mitochondrial damage through the cGAS‒STING pathway, thereby triggering type I IFN response to induce cell premature senescence and phenotypic switching that leads to the loss of fibrous cap, fibrous cap thinning, and plaque vulnerability. In contrast, attenuation of type I IFN response remarkably mitigated plaque vulnerability (Bi et al., 2021). These findings reveal that the cGAS‒STING pathway-mediated type I IFN response in VSMCs is essential for the pathogenesis of CKD-associated plaque vulnerability. Thus, alleviating type I IFN response may hold promise for the treatment of CKD-associated cardiovascular diseases. APOL1 risk alleles G1 and G2, which are associated with faster progress to lupus nephritis-associated end-stage renal disease (LN-ESRD) in African Americans, have been reported to be upregulated by the activation of the cGAS‒STING pathway and IFN signaling in human immortalized podocytes (Davis et al., 2019), suggesting a potential link between the cGAS‒STING pathway and LN-ESRD. All together, these results demonstrate that activation of the cGAS‒STING pathway contributes to kidney injury, uncovering potential new therapeutic targets for preventing the progression of AKI and CKD.

## Concluding remarks

Though significant progress has been made over the past several years in understanding the role of the cGAS‒STING signaling pathway in cytosolic DNA sensing and host defense (Motwani et al., 2019), emerging evidence strongly suggests that this pathway may also have functions beyond DNA sensing in innate immunity (Figure 2). In line with this view, dysregulation of cGAS and/or STING in adipocytes (Bai et al., 2017, 2020), hepatocytes (Luo et al., 2018; Luther et al., 2020), and human RPTECs (Maekawa et al., 2019) contributes to various physiological or pathophysiological consequences, such as insulin resistance (Bai et al., 2017; Bai and Liu, 2019), obesity (Bai et al., 2020), alcoholic and nonalcoholic liver diseases (Luo et al., 2018; Luther et al., 2020), as well as kidney dysfunction (Maekawa and Inagi, 2019; Figure 2). Further investigations would provide important new insight into the metabolic and renal functions of cGAS‒STING signaling components and their mechanisms of action in these biological processes.

Increasing evidence suggests a close and dynamic link between the immune and other biological systems in our body (Zmora et al., 2017). The crosstalks between immune cells and their neighboring cells in a given tissue establish a microenvironment that is pivotal for the adaptation to ever-changing environmental and nutritional statuses. Recent studies suggest that, in addition to its canonical innate immune function as a dsDNA sensor in host defense, the cGAS‒STING signaling pathway also plays a critical role in modulating metabolic and other biological processes (Bai and Liu, 2019). However, the precise mechanisms by which the cGAS‒STING signaling pathway regulates the metabolic processes remains obscure. The cGAS‒STING pathway is highly enriched in immune cells in metabolic tissues including the liver and adipose tissue, and activation of this pathway may promote inflammation, leading to a detrimental microenvironment in the tissues that causes insulin resistance or hepatosteatosis (Bai et al., 2017; Luo et al., 2018; Bai and Liu, 2019; Wang et al., 2020b). Given that the cGAS product 2ʹ3ʹ-cGAMP could be transferred from damaged cells to neighboring cells via gap junctions (Patel et al., 2009; Ablasser et al., 2013; Luther et al., 2020), it is also possible that activation of the cGAS‒STING pathway in tissue-resident immune cells may affect tissue homeostasis and function via a direct crosstalk between the immune cells and metabolic cells in the tissue.

Recent studies reveal that cGAS is localized not only in the cytosol but also at plasma membrane (Barnett et al., 2019) and in the nucleus (Bai and Liu, 2021), where it is associated with distinct nuclear substructures such as nucleosomes (Zierhut et al., 2019; Boyer et al., 2020; Kujirai et al., 2020; Michalski et al., 2020; Pathare et al., 2020; Wang et al., 2020a; Zhao et al., 2020), DNA replication forks (Chen et al., 2020), double-stranded breaks (Liu et al., 2018), and centromeres (Gentili et al., 2019). The nuclear localized cGAS shows much less or no activation, indicating an innate immune-independent role of cGAS in the nucleus. cGAS‒STING signaling has also been found to be associated with autophagy (Watson et al., 2012; Liang et al., 2014a, b; Lei et al., 2018), mitophagy (Newman and Shadel, 2018), apoptosis (White et al., 2014; Diner et al., 2016; Lum et al., 2018), and mTOR signaling (Yu et al., 2015; Hasan et al., 2017) under various cellular conditions. However, it remains to be determined whether the distinct cellular localization and distinctive functions of cGAS play a role in regulating metabolism.

In conclusion, despite a great advancement on our understanding of the role of the cGAS‒STING signaling pathway in innate immunity, a number of outstanding questions remain to be addressed. Given that many of the *in vivo* studies were done by using global cGAS or STING knockout mice, it remains to be determined as to how much of the observed phenotypes is due to a direct effect of cGAS or STING signaling in a given cell/tissue as opposed to a secondary effect. It is also unclear how the cGAS‒STING signaling pathway is specifically regulated in distinct cells or tissues. Furthermore, dysregulation of the cGAS‒STING pathway has been implicated in a spectrum of pathological conditions, but what is the connection between cGAS and/or STING dysregulation and metabolic dysfunction, cancer, autoimmune diseases, neurodegenerative disorders, and aging? It is likely that our knowledge will be greatly improved with the phenotype characterization of cell-specific cGAS or STING knockout animal models and with the development of more specific pharmacological agents targeting components in the cGAS‒STING signaling pathway. Understanding the tissue/cell-specific roles of the cGAS‒STING signaling pathway will provide novel and accurate insights into the mechanisms linking the immune and other biological systems in health and diseases.

## Funding

This work was supported in part by grants from the National Natural Science Foundation of China (NSFC; 81730022 and 81870601) and Innovative Basic Science Awards of American Diabetes Association (1-19-IBS-147).

**Conflict of interest:** none declared.
